# Supplementary figures and images for: Host species is linked to pathogen genotype for the amphibian chytrid fungus (Batrachochytrium dendrobatidis)
Source: PLoS One. 2022 Mar 14;17(3):e0261047. doi: 10.1371/journal.pone.0261047 (PMC8920232; doi:10.1371/journal.pone.0261047)

Figure S1

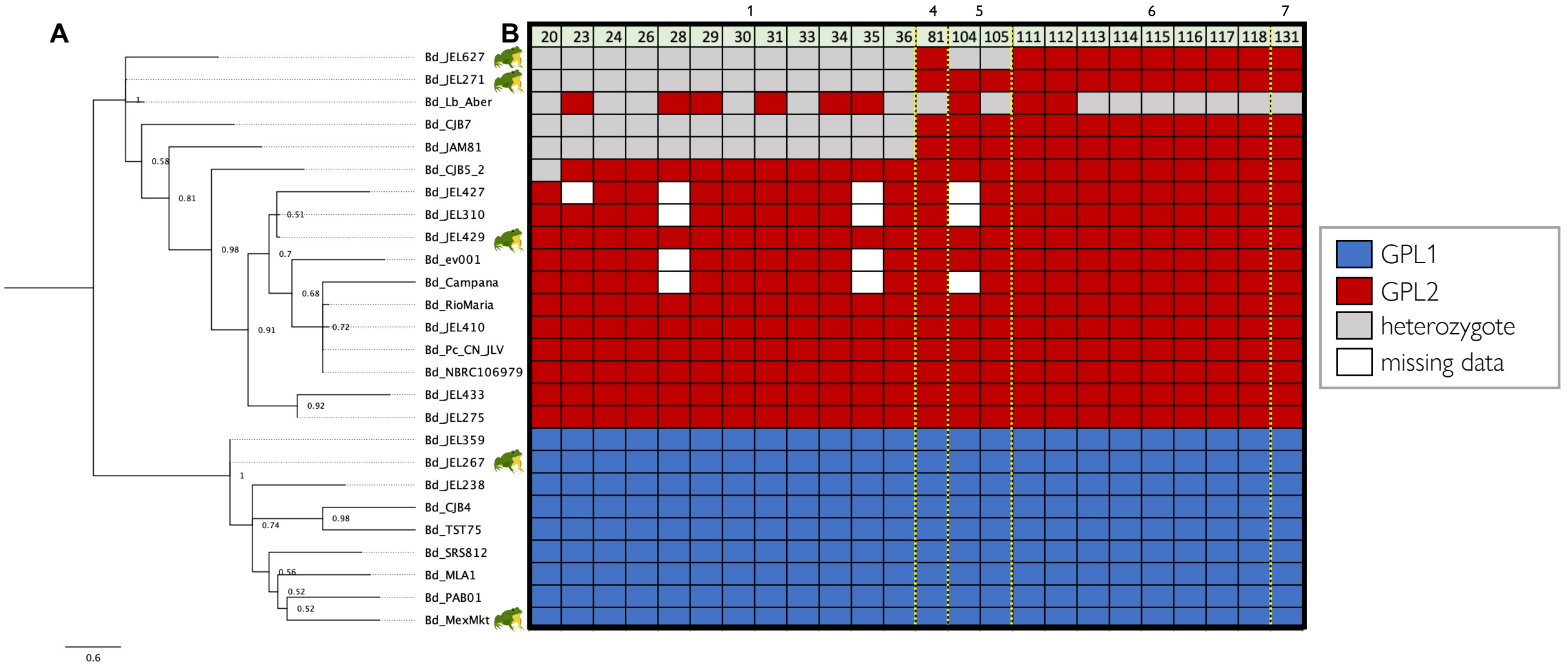

Figure S2

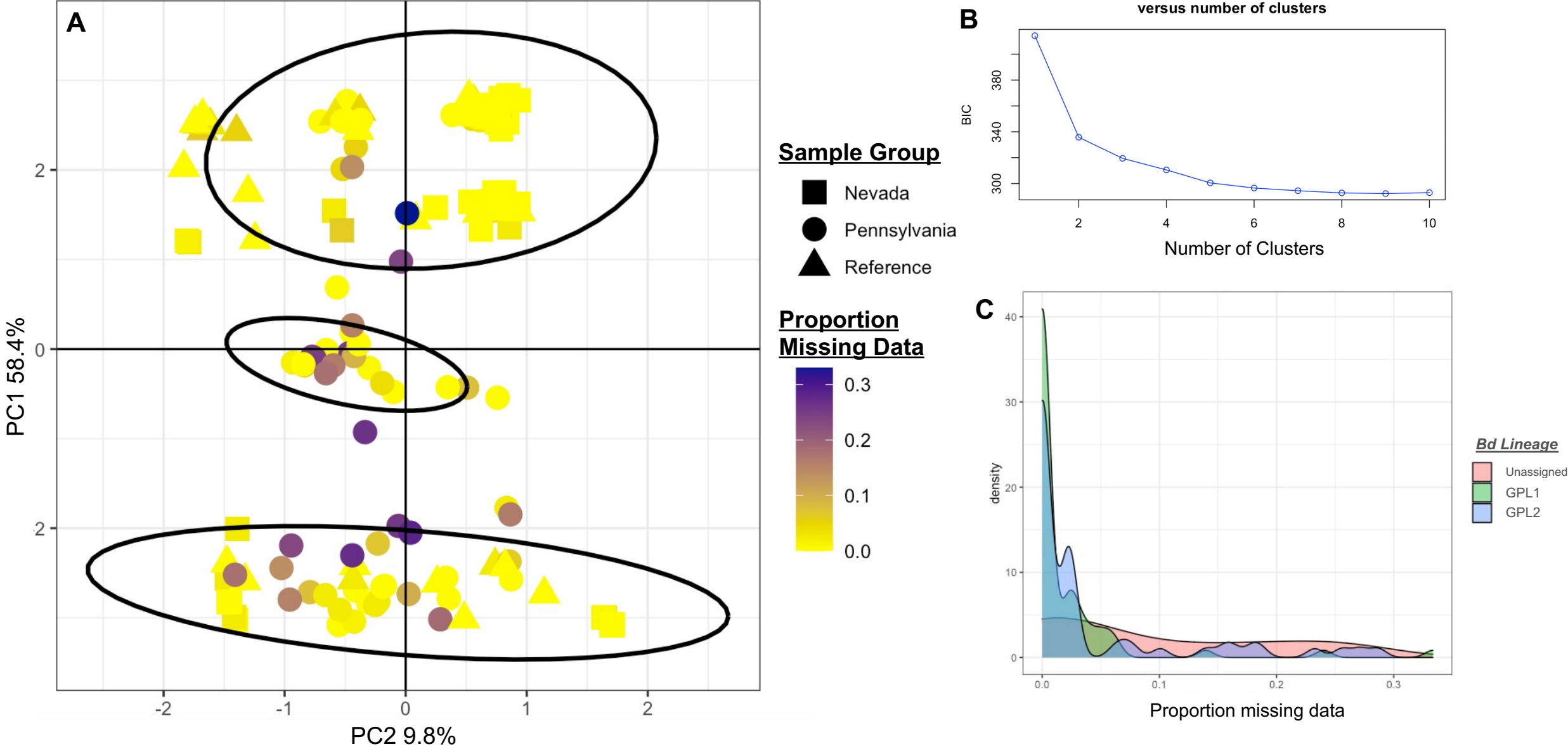

Figure S3

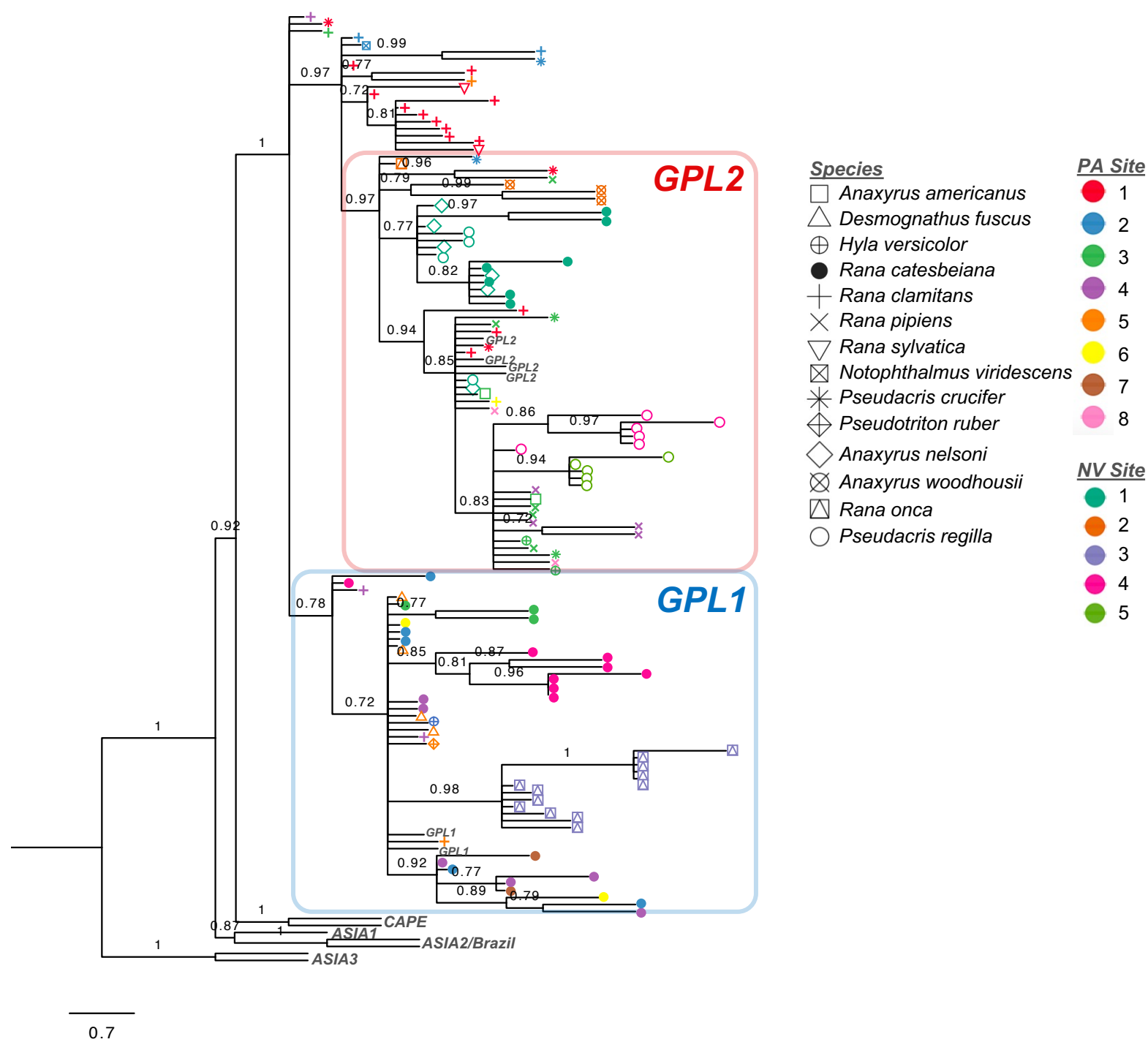

Figure S4

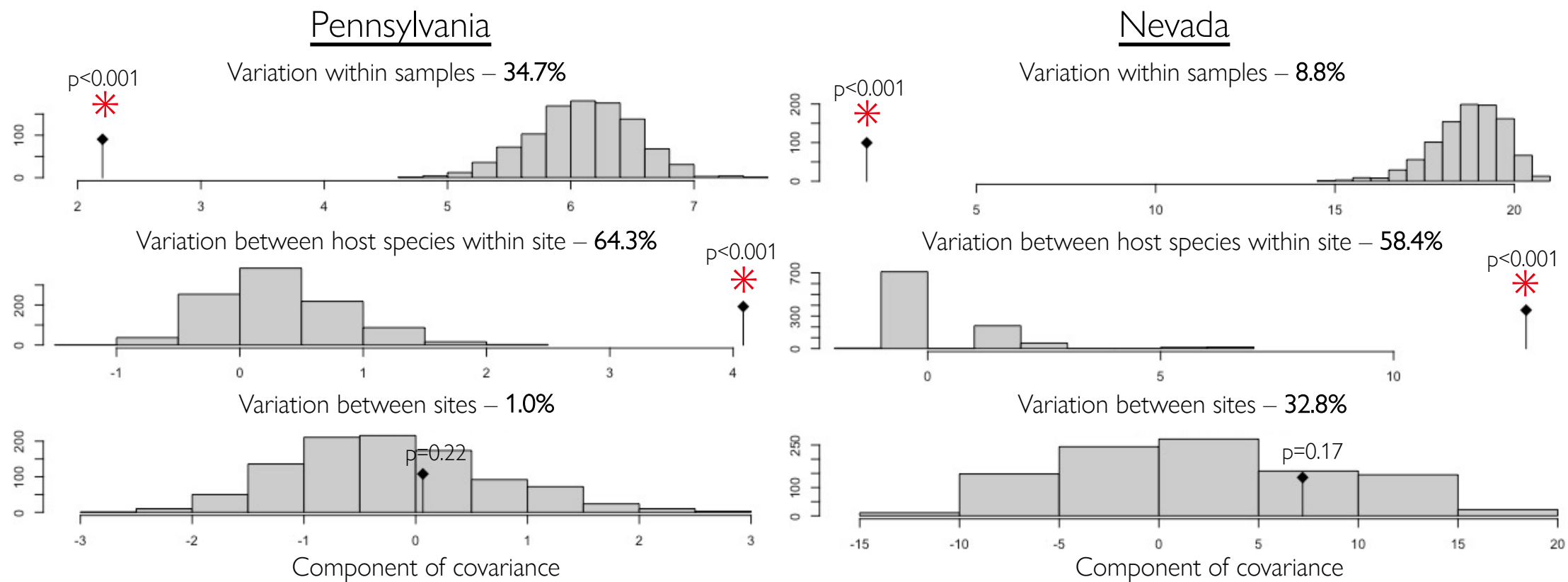

Figure S5

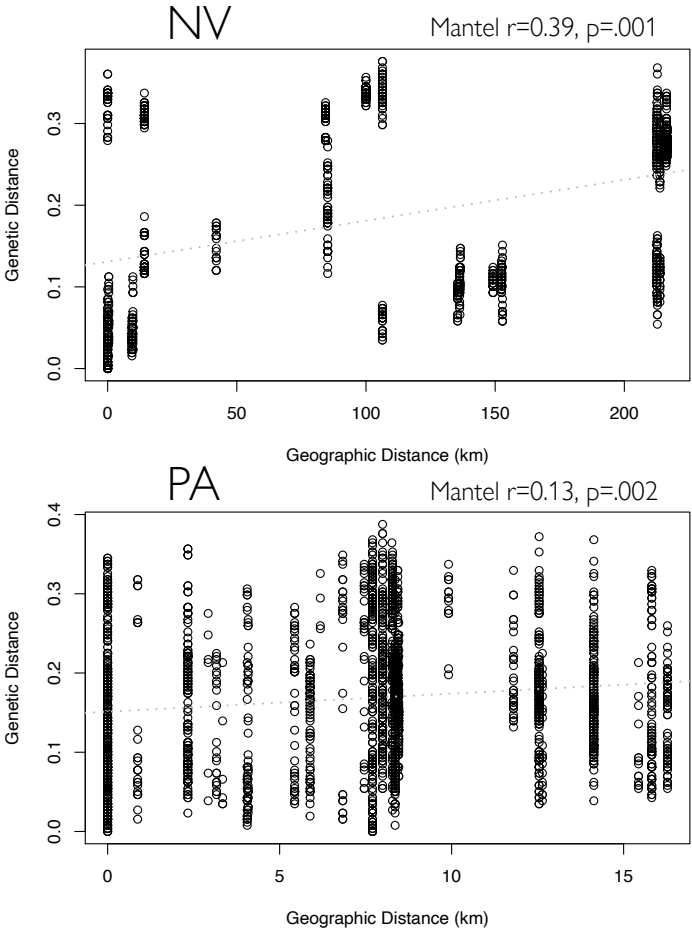

Figure S6

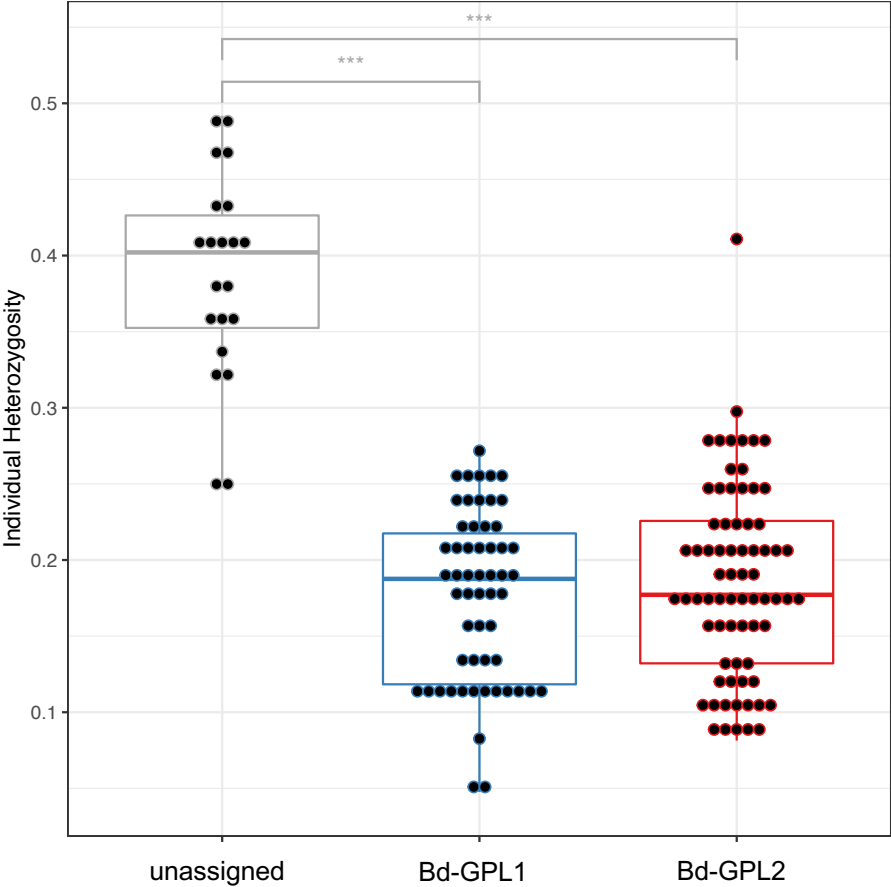

Supplement: S1 File — (PDF) [file pone.0261047.s001.pdf]
